# Proteomics and metabolomics analysis reveal potential mechanism of extended-spectrum β-lactamase production in *Escherichia coli*†

**DOI:** 10.1039/d0ra04250a

**Published:** 2020-07-17

**Authors:** He Ma, Bingjie Lai, Yufen Jin, Chang Tian, Jiaying Liu, Ke Wang

**Affiliations:** Department of Respiratory Medicine, The Second Hospital of Jilin University Changchun China kewangm1@hotmail.com; Department of Anesthesiology, The Second Hospital of Jilin University Changchun China; Department of Intensive Care Unit, The Second Hospital of Jilin University Changchun China; Clinical Laboratory, The Second Hospital of Jilin University Changchun China

## Abstract

In this study, ten clinical susceptible strains and ten clinical ESBL-EC (extended-spectrum β-lactamase-producing *Escherichia coli*) were screened and obtained by microbial identification using ITEK® 2 Compact. TMT (Tandem Mass Tag) proteomics analysis discovered 1553 DEPs (differentially expressed proteins) between ESBL-EC and non-ESBL-EC. In addition, an untargeted metabolomics assay by using UHPLC-MS (ultra-high-performance liquid chromatography-mass spectrometry) was applied to compare the differential profiles of metabolites between β-lactam antibiotic-sensitive *E. coli* and multidrug-resistant ESBL-producing *E. coli* strains. The PCA (principal component analysis) score plots and OPLS-DA (orthogonal projections to latent structures discriminant analysis) plots clearly discriminated ESBL-EC and non-ESBL-EC, and volcano analysis presented 606 and 459 altered metabolites between ESBL-EC *vs.* non-ESBL-EC in positive and negative ion modes, respectively. Interestingly, the bioinformatics analysis demonstrated that the purine metabolism pathway was enriched in ESBL-EC. These results suggest that the existence of extended-spectrum β-lactamase affects the metabolite and protein profiles of *E. coli*. The correlation analysis of metabolomics and proteomics data established a correlation between DEPs and differential metabolites in the purine metabolism pathway. Moreover, three metabolite candidates in the purine metabolism pathway were validated by the UPLC-MRM-MS (ultra-performance liquid chromatography multiple reaction monitoring mass spectrometry) method. Our data suggest that these DEPs and differential metabolites may play important roles in the antibiotic resistance of ESBL-EC. Our study can provide scientific data for the mechanism study of antibiotic resistance of ESBL-EC at the metabolite and protein levels and targeting modulators to these pathways may be effective for treatment of ESBL-EC strains.

## Abstract 2

Proteomic and metabolomics revealed the underlying mechanism of extended-spectrum β-lactamase production in *Escherichia coli*.

## Graphical abstract

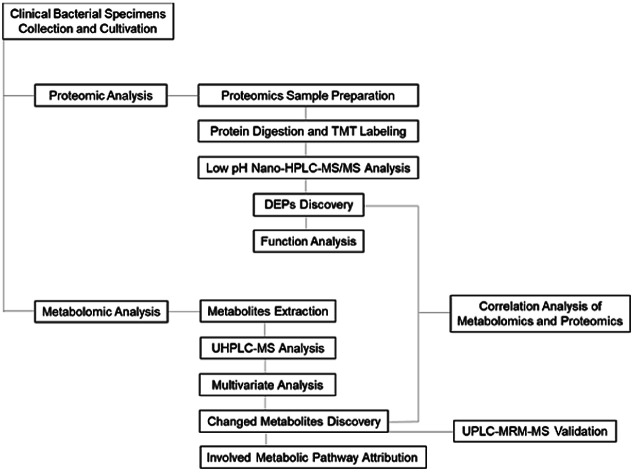

## 1. Introduction

*E. coli* (*Escherichia coli*) has the characteristics of being both a widespread gut commensal of vertebrates and a versatile pathogen.^1^*E. coli* is a common cause of urinary tract infection and intra-abdominal infection in humans of all ages,^2^ and it kills more than 2 million humans annually in the form of intra- and extra-intestinal diseases.^3,4^ ESBLs (Extended-spectrum β-lactamases) are plasmid-encoded β-lactamases resistant to penicillins, cephalosporins, β-lactams, and other antimicrobials, such as aminoglycosides and fluoroquinolones.^5^ The emergence of multidrug-resistant bacteria renders the treatment of bacterial infections an urgent global challenge.^6^ Thus, the mechanisms of *E. coli* resistance should be clearly understood in the future development of antibiotics, and new therapy methods should be sought.

Detecting and identifying metabolome-scale changes in microorganisms is essential in understanding their roles in cellular processes. Metabolites may be used to realize the molecular basis for a biological phenomenon, including cell signaling.^7^ Metabolomics, one of the most powerful and promising tools for analyzing metabolism,^8^ is a high-throughput analytical technology used to identify and quantify small amounts of metabolites.^9^ This tool provides a broad range of information on integrated cellular response.^10^ Furthermore, metabolomics has been applied to a variety of pathophysiological process, including cancer and diabetes, with the goal of identifying the biomarkers predictive of a disease onset and ensuring treatment efficacy monitoring.^11^ Studies have been reported on metabolomics techniques that focus on the role of bacterial metabolism in constraining the evolution of antibiotic resistance.^12^ Wei *et al.* characterized the mechanisms of *p*-aminosalicylic acid resistance in *Mycobacterium tuberculosis* might be associated with the increased metabolites level in phenylalanine metabolism pathway using multi-omics (genome, proteome, and metabolome) analysis.^13^ Zhao *et al.* used multi-omics (LC-MS based metabolomic, and iTRAQ labeling proteomic) analysis to assess capreomycin resistance on tlyA deficient and point mutation (G695A) *Mycobacterium tuberculosis* strains, with the finding that the greater drug tolerance of CAPr1 strains may be associated with the weakening of *S*-adenosyl-l-methionine-dependent methyltransferase activity and abnormal membrane lipid metabolism.^14^

In addition to metabolomics, proteomics is also a potent tool for improving the characterization of complex biological systems or pathologies. Proteomics methods have been used to identify protein network alterations, thus helping researchers understand the responses to various diseases.^15,16^ Protein expression studies based on proteomics have unique advantages, such as large-scale, high-throughput, high-sensitivity, and dynamic formats.^17^ The proteomics methods for several types of drug resistance have attracted increasing attention in previous studies.^18^ Investigating metabolome-scale changes may provide insights into the widespread modes of acquired bacterial resistance in clinics.^7^

In this study, a conjoint analysis of TMT (Tandem Mass Tag) proteomics and metabolomics was applied to compare the differential profile of proteins and metabolites between β-lactam antibiotic-sensitive *E. coli* and multidrug-resistant ESBL-producing *E. coli* strains and reveal the mechanisms of antibiotic resistance of clinical *E. coli* strains. The novelty of this study lies in revealing the metabolic characteristics of enzyme-producing bacteria and finding potential important pathways and genes for regulating enzyme production.

## 2. Materials and methods

### 2.1. Chemicals and reagents

Ultrapure water was produced by a Ming Che D24 UV water purification system (Merck Millipore, Burlington, USA). MeOH, ACN, NH_4_OAc, and NH_4_OH (CNW Technologies, Düsseldorf, Germany) were all MS grade. 2-Chloro-l-phenylalanine (Sigma, St. Louis, MO, USA) was used as an internal standard. HEPES, SDS, TFA, triethylammonium bicarbonate (TEAB), ammonium formate, Columbia agar, MacConkey agar, and Chocolate agar were obtained from Sigma (St. Louis, MO, USA). Dithiothreitol (DTT), iodoacetamide (IAA), and trypsin (sequencing grade) were obtained from Promega Life Sciences (Madison, USA). Urea was provided by GibcoBRL (MD, USA). EDTA and PMSF were obtained from Amesco (USA). Ethanol, formic acid, acetone, BCA protein quantification kit, TMT 6 standard kit, Acclaim PepMap C18 Column (100 μm × 20 mm, 75 μm × 250 mm) were purchased from Thermo Fisher Scientific (Waltham, MA, USA). TEAB and 25% ammonia were obtained from Santa Cruz (CA, USA). Sep Pak C_18_ cartridges (1 cc, 100 mg) and the Acquity UPLC®BEH C18 column (50 mm × 2.1 mm, 1.7 μm) were obtained from Waters (Milford, USA). The Luria–Bertani (LB) broth and LB agar were purchased from Becton Dickinson (Sparks, MD, USA). The lysis buffer for protein analysis was obtained from Roche Ltd (Basel, Switzerland). All other reagents were of ACS reagent grade.

### 2.2. Clinical bacterial specimens collection and cultivation

Clinical specimens of human phlegm, urine, blood, secretions were randomly collected on January–June 2018 from patients of the Second Hospital of Jilin University, the specimens were divided as experimental group (*n* = 10) and control group (*n* = 10). Detailed sample information was list in Table 1. The patients signed informed consent forms for the experimental study. All experimental procedures were performed in accordance with the Guidelines for the Collection and Application of Human Related Specimens of the Second Hospital of Jilin University and approved by the Ethics Committee of the Second Hospital of Jilin University. The age and gender of all patients included in this research were recorded. The phlegm samples must comply with the requirements of more than 25 leukocytes and less than 10 epithelial cells in the low-power field. The phlegm and urine samples were planted with Columbia agar with blood solution, MacConkey agar, and Chocolate agar plates and kept in a 5% CO_2_ incubator at 35 °C. Blood samples were cultured in automated blood culture flask system. The blood samples with bacterial infection were used for further plate culture. A single colony was selected and cultured in the LB broth to 0.5 MCF. After microbial identification and sensitivity analysis with the aid of VITEK® 2 Compact, the bacterial suspension was coated on a plate, then ceftazidime, ceftazidime/clavulanic acid and cefotaxime, and cefotaxime/clavulanic acid were used as indicators to detect the ESBLs in the *E. coli* according to the CLSI (2014) Performance Standards for Antimicrobial Susceptibility Testing.

**Table 1:** The paralleled collection times for clinical bacterial specimens

Sample number	Experimental group	Collection time	Gender	Control group	Collection time	Gender
1	Urine	2018/1/25	Male	Urine	2018/2/15	Male
2	Blood	2018/3/1	Male	Phlegm	2018/3/9	Female
3	Blood	2018/3/12	Female	Phlegm	2018/3/21	Female
4	Phlegm	2018/3/29	Male	Blood	2018/3/27	Male
5	Phlegm	2018/4/20	Female	Blood	2018/4/1	Female
6	Blood	2018/5/3	Female	Urine	2018/4/27	Male
7	Urine	2018/5/12	Male	Blood	2018/5/6	Male
8	Phlegm	2018/5/20	Female	Urine	2018/5/24	Female
9	Urine	2018/5/21	Female	Phlegm	2018/6/1	Male
10	Vaginal secretion	2018/5/27	Female	Phlegm	2018/6/5	Female

### 2.3. Proteomics analysis

#### 2.3.1. Proteomics sample preparation

Each sample was mixed with 1 mL of lysis buffer and subjected to sonication on ice for 10 min. After centrifugation at 20 000×*g* for 30 min at 4 °C, the supernatant was collected and measured for protein concentration with BCA. Then, the 100 μg protein was selected and set to the constant volume of 100 μL per test with 100 mM of TEAB.

#### 2.3.2. Protein digestion and TMT labeling

Each sample was co-incubated with 5 μL of DTT (200 mM) for 1 h at 55 °C, followed by 30 min for the RT co-incubation of 5 μL of IAA (375 mM). After the precipitation with cold acetone, the sample was dissolved with 100 μL of TEAB and subjected to trypsin digestion. The digested peptide product was labeled using TMT kits. The sample was ready for separation after desalting and vacuum drying.

#### 2.3.3. Low pH nano-HPLC-MS/MS analysis and data processing

After high pH reverse phase separation of the sample, the fractions were resuspended with 40 μL solvent C respectively (C: water with 0.1% formic acid; D: ACN with 0.1% formic acid), separated by nanoLC and analyzed by on-line electrospray tandem mass spectrometry. The experiments were performed on a Nano Aquity UPLC system (Waters Corporation, Milford, MA) connected to a quadrupole-Orbitrap mass spectrometer (Q-Exactive) (Thermo Fisher Scientific, Bremen, Germany) equipped with an online nano-electrospray ion source. 4 μL peptide sample was loaded onto the trap column (Thermo Scientific Acclaim PepMap C18, 100 μm × 2 cm), with a flow of 10 μL min^−1^ for 3 min and subsequently separated on the analytical column (Acclaim PepMap C18, 75 μm × 25 cm) with a linear gradient, from 5% D to 30% D in 80 min. The column was cleaned then was re-equilibrated at initial conditions for 10 min. The column flow rate was maintained at 300 nL min^−1^ and column temperature was maintained at 45 °C. The electrospray voltage of 2.0 kV *versus* the inlet of the mass spectrometer was used. The Q-Exactive mass spectrometer was operated in the data-dependent mode to switch automatically between MS and MS/MS acquisition. Survey full-scan MS spectra (*m*/*z* 350–1600) were acquired with a mass resolution of 70 K, followed by fifteen sequential high energy collisional dissociation (HCD) MS/MS scans with a resolution of 17.5 K. In all cases, one microscan was recorded using dynamic exclusion of 30 seconds. MS/MS fixed first mass was set at 100. Tandem mass spectra were then subjected to the proteome database searching and quantitative data analysis as described in the ESI.†^19^

### 2.4. Metabolomics analysis

#### 2.4.1. Metabolites extraction

After the centrifugation of the bacterial culture solution at 3000×*g* for 15 min at 4 °C, the 50 mg pellets were taken and suspended in 1000 μL of the cold extraction solution (40% MeOH, 40% acetic acid, and 20% H_2_O) containing an internal standard (2-chloro-l-phenylalanine). The suspension was homogenized with a ball mill at 40 Hz for 5 min and subjected to 5 min of ultrasonic water bath, which was repeated three times, then the protein was precipitated by incubation for 1 h at −20 °C. After centrifugation at 3000×*g* for 15 min at 4 °C, the 825 μL supernatant was collected and vacuum-dried. The dried metabolite sample was reconstituted with 100 μL of the extraction solution (50% ACN and 50% H_2_O), vortexed for 30 s, subjected to 10 min of ultrasonic water bath at 4 °C, and centrifuged at 3000×*g* for 15 min at 4 °C. The 60 μL supernatant was collected and prepared for UHPLC-QTOF-MS analysis. The 10 μL supernatant was collected from each sample and combined as the QC sample.

#### 2.4.2. UHPLC-MS (ultra-high-performance liquid chromatography-mass spectrometry) analysis

For the untargeted metabolomics analysis, UHPLC-MS analyses were performed using an UHPLC system (1290, Agilent Technologies) with a UPLC BEH Amide column (1.7 μm, 2.1 × 100 mm, Waters) coupled to Triple TOF 5600 (Q-TOF, AB Sciex). The mobile phase consisted of 25 mM NH_4_OAc and 25 mM NH_4_OH in water (pH = 9.75) (A) and acetonitrile (B) was carried with elution gradient as follows: 0 min, 95% B; 7 min, 65% B; 9 min, 40% B; 9.1 min, 95% B; 12 min, 95% B, which was delivered at 0.5 mL min^−1^. The injection volume was 2 μL. The Triple TOF mass spectrometer was used for its ability to acquire MS/MS spectra on an information-dependent basis (IDA) during an LC/MS experiment. In this mode, the acquisition software (Analyst TF 1.7, AB Sciex) continuously evaluates the full scan survey MS data as it collects and triggers the acquisition of MS/MS spectra depending on preselected criteria. In each cycle, 12 precursor ions whose intensity greater than 100 were chosen for fragmentation at collision energy (CE) of 30 V (15 MS/MS events with product ion accumulation time of 50 m s each). ESI source conditions were set as following: ion source gas 1 as 60 psi, ion source gas 2 as 60 psi, curtain gas as 35 psi, source temperature 650 °C, Ion Spray Voltage Floating (ISVF) 5000 V or −4000 V in positive or negative modes, respectively. Untargeted metabolomics were then subjected to the data processing and metabolite annotation as described in the ESI.†^20,21^

#### 2.4.3. UPLC-MRM-MS (ultra-performance liquid chromatography multiple reaction monitoring mass spectrometry) analysis

For the targeted metabolomics analysis, the UHPLC separation was carried out using an Agilent 1290 Infinity II series UHPLC System (Agilent Technologies), equipped with a Waters ACQUITY UPLC BEH Amide column (100 × 2.1 mm, 1.7 μm). The mobile phase A was 1% formic acid in water, and the mobile phase B was acetonitrile. The elution gradient was shown in Table S6.† The column temperature was set at 35 °C. The auto-sampler temperature was set at 4 °C and the injection volume was 1 μL. An Agilent 6460 triple quadrupole mass spectrometer (Agilent Technologies), equipped with an AJS electrospray ionization (AJS-ESI) interface, was applied for assay development. Typical ion source parameters were: capillary voltage = +4000/−3500 V, nozzle voltage = +500/−500 V, gas (N_2_) temperature = 300 °C, gas (N_2_) flow = 5 L min^−1^, sheath gas (N_2_) temperature = 250 °C, sheath gas flow = 11 L min^−1^, nebulizer = 45 psi. The MRM parameters for each of the targeted analytes were optimized using flow injection analysis, by injecting the standard solutions of the individual analytes, into the API source of the mass spectrometer. Several most sensitive transitions were used in the MRM scan mode to optimize the collision energy for each Q1/Q3 pair (Table S7†). Among the optimized MRM transitions per analyte, the Q1/Q3 pairs that showed the highest sensitivity and selectivity were selected as ‘quantifier’ for quantitative monitoring. The additional transitions acted as ‘qualifier’ for the purpose of verifying the identity of the target analytes. Agilent MassHunter Work Station Software (B.08.00, Agilent Technologies) was employed for MRM data acquisition and processing. Calibration curves LOD (limit of detection) and LOQ (limit of quantitation), precision and accuracy were established to guarantee the analysis stability, details see in the ESI.†

### 2.5. Statistical analysis

The data were analyzed with student's *T* test by using Excel 2013. A *p*-value of <0.05 was considered significant.

## 3. Results

### 3.1. TMT quantitative proteomics analysis

**Fig. 1:**
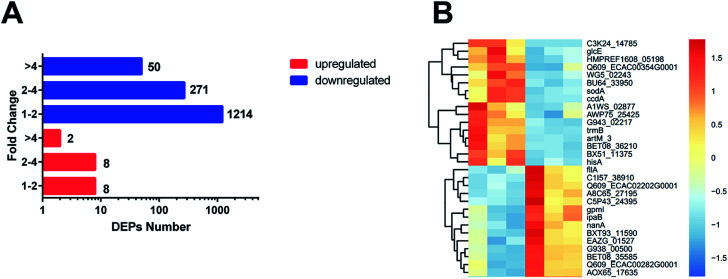
(A) Number of proteins identified as upregulated and downregulated between ESBL-EC and non-ESBL-EC. The red bars represent the upregulated proteins. The blue bars represent the downregulated proteins. The *x*-axis represents the number of DEPs. The *y*-axis represents the fold change value. (B) Heatmap based on representative hierarchical clustering analysis with 30 DEPs between ESBL-EC and non-ESBL-EC (the detailed heatmap is shown in Fig. S1†). The relative protein level is depicted in color scale, in which red indicates upregulation, and blue indicates downregulation.

The 1553 DEPs (differentially expressed proteins) (fold change < 0.83 or fold change > 1.2; *p*-value < 0.05) were identified by TMT MS; among the DEPs, 18 were upregulated and 1535 were downregulated in the ESBL-EC (Fig. 1A, B and S1†). The detailed results of the DEPs are shown in Table S1.†

### 3.2. Function analysis results

**Fig. 2:**
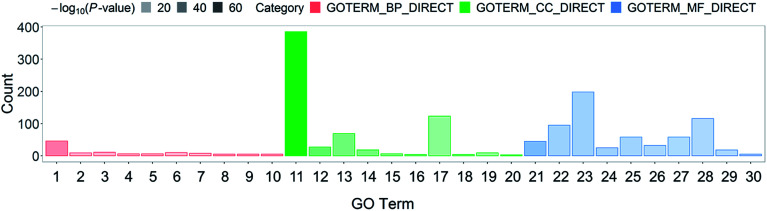
Top 10 GO classification histograms of DEPs for ESBL-EC *vs.* non-ESBL-EC. The *x*-axis represents GO_BP (GO Term Biological Process), GO_CC (Cellular Component), and GO_MF (Molecular Function). (1) Translation, (2) transcription antitermination, (3) glycolytic process, (4) isopentenyl diphosphate biosynthetic process, methylerythritol 4-phosphate pathway, (5) negative regulation of translational initiation, (6) nucleobase-containing small molecule interconversion, (7) ubiquinone biosynthetic process, (8) nucleotide phosphorylation, (9) terpenoid biosynthetic process, (10) DNA-templated transcription, termination, (11) cytosol, (12) cytosolic large ribosomal subunit, (13) membrane, (14) cytosolic small ribosomal subunit, (15) chromosome, (16) DNA topoisomerase complex (ATP-hydrolyzing), (17) cytoplasm, (18) glycerol-3-phosphate dehydrogenase complex, (19) cell division site, (20) cell surface, (21) structural constituent of ribosome, (22) identical protein binding, (23) protein binding, (24) rRNA binding, (25) zinc ion binding, (26) RNA binding, (27) magnesium ion binding, (28) ATP binding, (29) tRNA binding, (30) mRNA 5′-UTR binding. The *y*-axis represents the number of involved DEPs between ESBL-EC and non-ESBL-EC.

Systematic and integrative analysis was performed using DAVID to gain a comprehensive understanding of the DEPs.^22^ The properties of the genes and gene products of the DEPs were classified into three categories: GO_BP (GO Term Biological Process), GO_CC (Cellular Component), and GO_MF (Molecular Function). The classifications were used to identify the potential affected pathways. The DEPs were significantly enriched in 53 GO terms (*p*-value < 0.05). Among them, 29 were BP terms, 7 were CC terms, and 17 were MF terms (Table S2†). The 30 most representative and markedly enriched GO terms within the three main functional modules are shown in Fig. 1C. The leading seven BP terms were “translation”, “transcription antitermination”, “glycolytic process”, “isopentenyl diphosphate biosynthetic process, methylerythritol 4-phosphate pathway,” “negative regulation of translational initiation”, “nucleobase-containing small molecule interconversion” and “Ubiquinone biosynthetic process” (Fig. 2).

In addition, significantly enriched metabolic pathways of DEPs were identified using the KEGG database to determine the systemic difference between ESBL-EC and non-ESBL-EC.^23^ A total of 82 significant systemically changed KEGG pathways were detected (Table S3†). PPI network analyses are important in the discovery of the variation tendency of DEPs in proteomics analysis, and they are helpful in targeting the key node between DEPs.

**Fig. 3:**
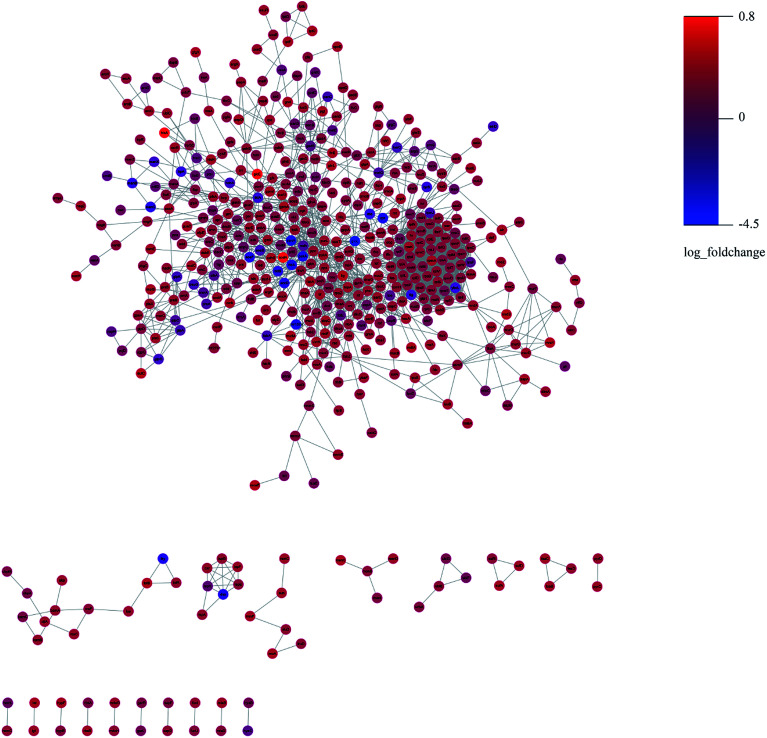
PPI (protein–protein interaction) network of DEPs between ESBL-EC and non-ESBL-EC. Large sizes and dark colors represent the high impact and level of DEPs.

We constructed the PPI of *E. coli* by using the STRING database^23^ to uncover the potential protein interaction of DEPs and the biological functions in the bacterial cellular processes for ESBL-EC (Fig. 3). Moreover, the PPI networks analysis was used to obtain different central nodes, including hisA, glcE, soda, and GroL.

### 3.3. Multivariate analysis of UPLC-QTof/MS data for the metabolomics study

**Fig. 4:**
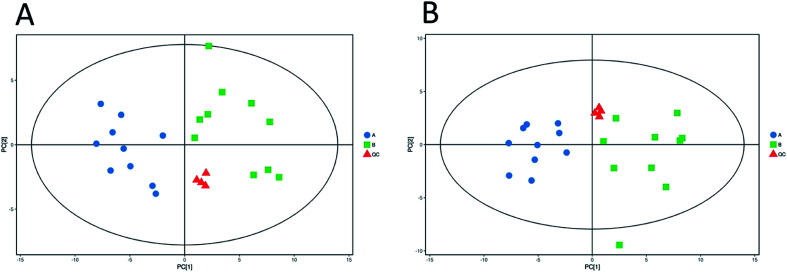
Multivariate analysis of untargeted metabolomics data. PCA score plots of metabolic profile from different groups in positive ESI mode (A) and negative ESI mode (B).

Unsupervised pattern recognition PCA (principal component analysis) was conducted to visualize clearly the separation of the metabolites between ESBL-EC and non-SBL-EC (Fig. 4). QC injections were closely clustered in the PCA score plots in both positive mode (*R*^2^*X* = 0.525) and negative mode (*R*^2^*X* = 0.531). This study has verified the stability of the UPLC-QTof/MS system.

**Fig. 5:**
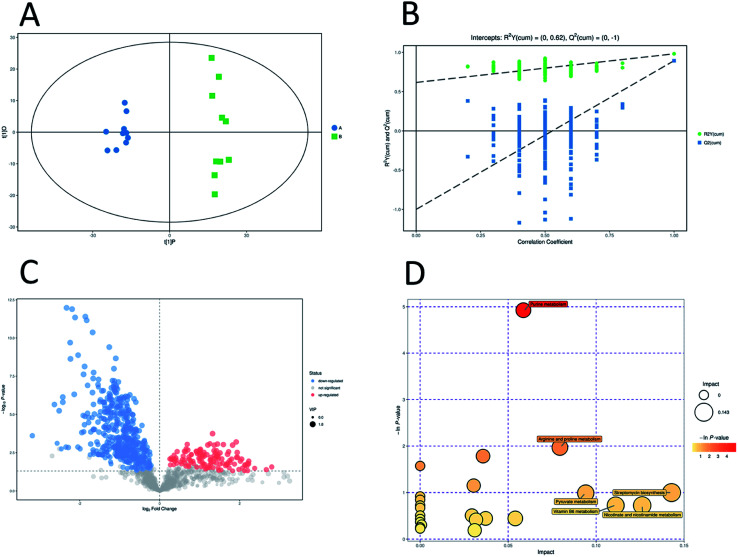
Multivariate analysis of untargeted metabolomics data and pathway analysis in positive mode. OPLS-DA score plots of metabolic profiling of different groups (A). The corresponding 200 random permutations plots of the OPLS-DA models (B). Volcano plots for ESBL-EC *vs.* non-ESBL-EC (C). The relative metabolite level is depicted according to color scale. Red represents the upregulation and blue represents the downregulation of the metabolites in ESBL-EC *vs.* non-ESBL-EC. Large sizes represent high VIP values of metabolites. Overview of significantly changed pathway analysis for ESBL-EC *vs.* non-ESBL-EC (D). The *x*-axis represents pathway enrichment. The *y*-axis represents the pathway impact. Large sizes and dark colors represent the major pathway enrichment and high pathway impact values, respectively.

**Fig. 6:**
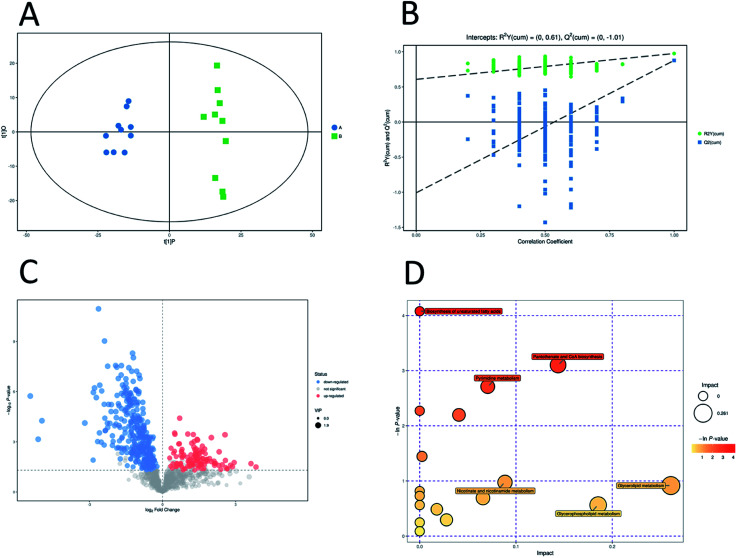
Multivariate analysis of untargeted metabolomics data and pathway analysis in negative mode. OPLS-DA score plots of metabolic profiling of different groups (A). The corresponding 200 random permutations plots of the OPLS-DA models (B). Volcano plots for ESBL-EC *vs.* non-ESBL-EC (C). The relative metabolite level is depicted according to color scale. Red represents the upregulation and blue represents the downregulation of the metabolites in ESBL-EC *vs.* non-ESBL-EC. Large sizes represent high VIP values of metabolites. Overview of significantly changed pathway analysis for ESBL-EC *vs.* non-ESBL-EC (D). The *x*-axis represents pathway enrichment. The *y*-axis represents the pathway impact. Large sizes and dark colors represent the major pathway enrichment and high pathway impact values, respectively.

In addition, OPLS-DA (orthogonal projections to latent structures discriminant analysis) was performed to remove the descriptor variables that were not correlated to the property variables and determine the differential metabolite profiles that was responsible for the classification between the two groups (Fig. 5A and 6A). The *R*^2^*X* and *R*^2^*Y* of the OPLS-DA model were 0.364 and 0.982 for the ESI+ mode and 0.352 and 0.976 for the ESI− mode, respectively. Subsequently, seven-fold cross validation was implemented and applied with 200 random permutations to validate the OPLS-DA models. All blue *Q*^2^ values at the left part were lower than the original points on the right part (Fig. 5B and 6B). This finding indicates that the original models are robust and have no overfitting.

### 3.4. Potential marker identification and the involved metabolic pathway attribution

The volcano plots, which were produced on the basis of OPLS-DA, depict the contribution of the variables between the two groups (Fig. 5C and 6C). MetaboAnalyst was used to search for the pathways of the changed metabolites. The pathway analysis based on the potential biomarkers showed 18 different metabolic pathways between the two groups (Fig. 5D, 6D; Tables 2 and 3).

**Table 2:** Pathway analysis of differential metabolites for ESBL-EC *vs.* non-ESBL-EC in positive mode

Pathway	Total	Hits	Raw *p*	−ln(*p*)	FDR	Impact
Purine metabolism	73	9	0.007253	4.9264	0.63099	0.05866
Arginine and proline metabolism	41	4	0.13937	1.9706	1	0.0794
Pyrimidine metabolism	44	4	0.1679	1.7844	1	0.03574
Glycerophospholipid metabolism	23	2	0.31626	1.1512	1	0.03043
Streptomycin biosynthesis	9	1	0.36767	1.0006	1	0.14286
Pyruvate metabolism	26	2	0.37182	0.98934	1	0.09409
Vitamin B6 metabolism	13	1	0.48491	0.72379	1	0.11111
Nicotinate and nicotinamide metabolism	13	1	0.48491	0.72379	1	0.12617
Butanoate metabolism	18	1	0.60186	0.50774	1	0.02941
Citrate cycle (TCA cycle)	20	1	0.64096	0.44479	1	0.0372
Propanoate metabolism	20	1	0.64096	0.44479	1	0.05405
Glutathione metabolism	21	1	0.65908	0.41692	1	0.03175
Valine, leucine and isoleucine biosynthesis	26	1	0.73704	0.30511	1	0.00085
Cysteine and methionine metabolism	34	1	0.82693	0.19004	1	0.03099

**Table 3:** Pathway analysis of differential metabolites for ESBL-EC *vs.* non-ESBL-EC in negative mode

Pathway	Total	Hits	Raw *p*	−ln(*p*)	FDR	Impact
Pantothenate and CoA biosynthesis	23	3	0.045	3.1011	1	0.14377
Pyrimidine metabolism	44	4	0.066497	2.7106	1	0.07066
Purine metabolism	73	5	0.11116	2.1967	1	0.04076
Pyruvate metabolism	26	2	0.23588	1.4444	1	0.00239
Nicotinate and nicotinamide metabolism	13	1	0.37763	0.97384	1	0.08859
Glycerolipid metabolism	14	1	0.40008	0.91609	1	0.26087
Peptidoglycan biosynthesis	17	1	0.50104	0.69108	1	0.06571
Glycerophospholipid metabolism	23	1	0.56972	0.56262	1	0.18536
Valine, leucine and isoleucine biosynthesis	26	1	0.6151	0.48597	1	0.0178
Galactose metabolism	37	1	0.745	0.29438	1	0.02786

### 3.5. Correlation analysis of metabolomics and proteomics

**Fig. 7:**
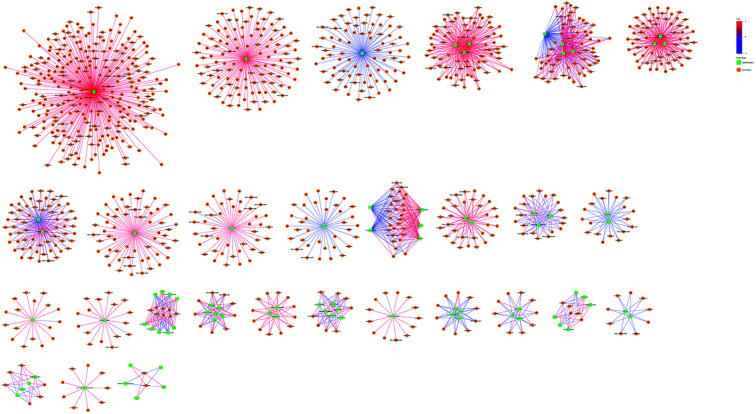
Metabolite–protein interaction network based on the differential metabolites and DEPs for ESBL-EC *vs.* non-ESBL-EC in positive mode. The green squares represent differential metabolites. The yellow circles represent differential protein genes. The red line represents a positive correlation between the differential metabolites and the differential protein genes. The blue line represents a negative correlation.

**Fig. 8:**
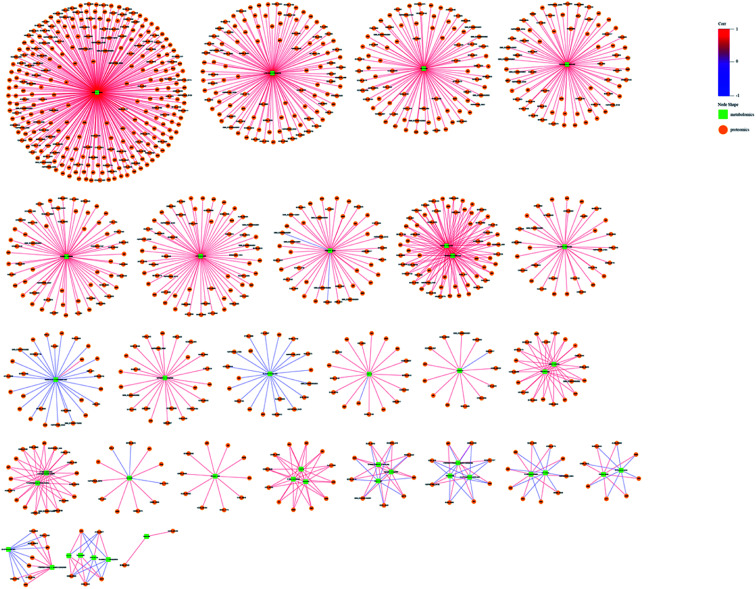
Metabolite–protein interaction network based on the differential metabolites and DEPs for ESBL-EC *vs.* non-ESBL-EC in negative mode. The green squares represent differential metabolites. The yellow circles represent differential protein genes. The red line represents a positive correlation between the differential metabolites and the differential protein genes. The blue line represents a negative correlation.

The omic-domain data integration for ESBL-EC was obtained by subjecting the content information of the DEPs and differential metabolites to relevant calculations by using the Spearman algorithm. The *p*-value of the correlation matrix was used for the follow-up analysis. A correlation network graph (Fig. 7 and 8) was constructed on the basis of the data that could meet both the correlation coefficient of >0.9 and the *p*-value of <0.05. In the figure, the green squares represent differential metabolites, the yellow circles represent the differential protein genes, the red line represents a positive correlation between the differential metabolites and the differential protein genes, and the blue line represents a negative correlation. Tables S8–S11† show all of the binary relations of the subnetworks.

### 3.6. Target metabolomics analysis

The drug resistance mechanism of ESBL-EC was further investigated, then the non-target metabolomics results were verified in this study. The sequential research was focused on the differential metabolites in the purine metabolism pathway (eco00230-*E. coli* K-12 MG1655). Three differential metabolites (2-deoxyadenosine monohydrate, 2,6-dihydroxypurine, and xanthosine) in the purine metabolism pathway were selected and subjected to target metabolomics analysis assay development by using UHPLC-QQQ-MS (MRM). As shown in Fig. S2,† the baseline separation of the metabolites can be successfully obtained, and the retention time and peak shapes of all of the analytes present a good match between the standard solution and the real sample, *i.e.*, 2-deoxyadenosine monohydrate (RT, 2.36 min), 2,6-dihydroxypurine (RT, 2.28 min), and xanthosine (RT, 3.30 min). Appropriate linear range with high linearity and low LOD and LOQ facilitate sensitive and selective analysis for the metabolites from biological samples. The LODs of the three metabolites were 1.22–19.53 nM, while the LOQs were 2.44–39.06 nM, with high linearity (*R*^2^ > 0.9997). The details of the calibration curves are shown in Table S4.† The five repeated injections presented a recovery from 93.5% to 104.2%, and the RSD of the targeted matabolites was less than 3.1% (Table S5†), which accords with the quantification of the targeted metabolomics analysis.

**Fig. 9:**
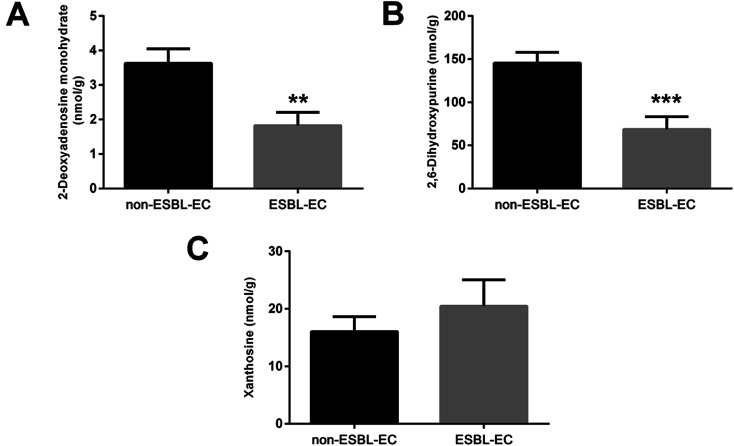
Target metabolomics analysis by using MRM for three differential metabolites for ESBL-EC *vs.* non-ESBL-EC: (A) 2-deoxyadenosine monohydrate, (B) 2,6-dihydroxypurine, and (C) xanthosine. The values are presented as mean ± SD, *n* = 10. **p-value < 0.01, and ***p-value < 0.001 and compared with the non-ESBL-EC group.

Then the rapid MRM based target metabolomics analysis was successfully applied to absolutely quantify the changed metabolites in the clinical bacterial samples. The MRM result (Fig. 9) showed that the contents of the 2-deoxyadenosine monohydrate and 2,6-dihydroxypurine were downregulated (*p*-value < 0.01). This finding is consistent with the non-target metabolomics analysis. However, the xanthosine was relatively overexpressed in ESBL-EC (*p*-value > 0.05), a finding inconsistent with the non-target metabolomics analysis.

## 4. Discussion

Antibiotics are commonly used as the first line of action in the treatment or prevention of certain bacterial infections.^24^ However, antibiotics do not work for patients infected with the ESBL-producing *E. coli* and thus a worldwide important public health concern.^25^ As discussed earlier, the antibiotic resistance and ESBL production capacity of *E. coli* remains poorly understood.^18^ Here, we examined for the first time the proteomics and metabolomics differences between ESBL-EC and non-ESBL-EC to clarify the potential mechanism of the ESBL-EC's antibiotic resistance and provide a scientific evidence for the discovery of novel targets for groundbreaking therapies.

Among the 18 different metabolic pathways classified between ESBL-EC and non-ESBL-EC, the purine metabolism pathway showed a high enriched degree, as characterized by the lowest ln *p*-value and dark bubble in the bubble plots. Purine metabolism was found to be correlated to the drug resistance of parasites and the drug resistance and tumor relapse of childhood ALL in previous work.^26,27^ More recently, a comparative metabolomics study based on GC-MS showed that the metabolic profiles of purine metabolism fluctuates in multi-drug-resistant *E. coli* strains; this result from previous work^18^ agrees with our current findings. In addition, in using metabolomics and proteomics correlation analysis, we found drug resistance affects the metabolite and protein profiles of *E. coli*, as many central metabolite nodes were obtained, including 2-deoxyadenosine monohydrate, 2,6-dihydroxypurine, and xanthosine, which have significant correlations with DEPs.

Considering the potential significance of purine metabolism for the drug resistance of ESBL-EC, target metabolomics analysis was performed using MRM. We selected three different metabolites (2-deoxyadenosine monohydrate, 2,6-dihydroxypurine, and xanthosine) in the purine metabolism pathway for further quantitative verification analysis, with comprehensive consideration of the MS2 score, *p*-value, VIP, and metabolite–protein interactions in the correlation analysis. The MRM analytical results coincided with the non-targeted metabolomics data, suggesting the reliability of our analytical method. Moreover, the above metabolites may be involved in the drug resistance of the bacteria in ESBL-EC.

DEPs may play important roles in the antibiotic resistance and ESBL production capacity of ESBL-EC. The present work has provided scientific data for the metabolic and protein level characteristics and involved important pathways of clinical β-lactamase enzyme-producing bacteria, which revealed the potential mechanism for the antibiotic resistance of ESBL-EC.

The main purpose of the experiment is using the proteomic and metabolomics to explore the underline mechanism of extended-spectrum β-lactamase production in *E. coli*. On the basic of above study, our future work is going on, such as performing site-by-point editing of the critical node protein-related genes or the purine metabolism pathway in ESBL-EC by using gene editing tools, such as the CRISPR/Cas9 system, based on the above research results. We also plan to analyze the bacterial virulence, extended-spectrum β-lactamase activity, and antibiotic resistance of genetically engineered *E. coli*. Another plan is to construct a gRNA recombination vector-based vaccine carrier bacteria with the aim of eliminating or weakening the activity of β-lactam drug resistance genes. We also intend to test the efficacy of the vaccine carrier bacteria *in vivo*. Briefly, mice will be pre-stimulated with the vaccine carrier bacteria, then we shall establish the mouse infection model by using clinical ESBL-EC, and finally, we will observe the therapeutic effect of β-lactam drugs on the infection of mice.

## 5. Conclusion

**Fig. 10:**
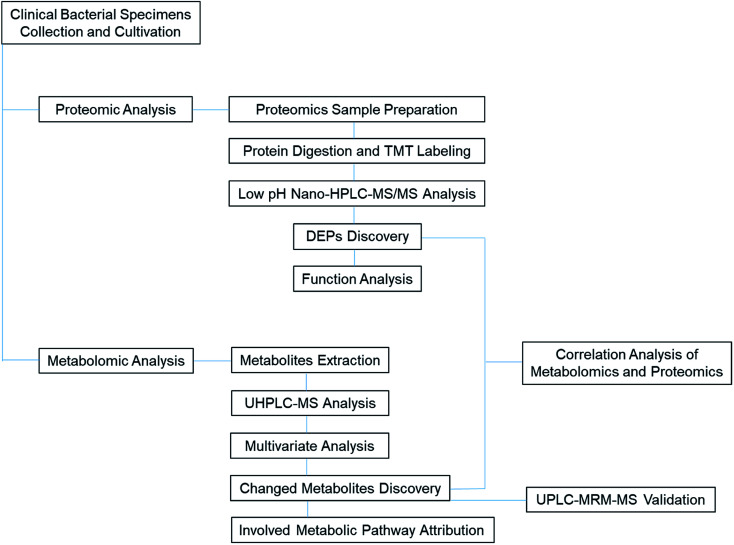
TOC graph for illustration of the proteomic and metabolomic analytical process of clinical ESBL specimens.

This basic research has shown how metabolic and proteomics alterations can manifest in ESBL-EC. The untargeted metabolomics result was also confirmed by the LC-MRM-MS analysis. With the aid of metabolomics and proteomics correlation bioinformatics analysis, we obtained many central metabolite nodes and their corresponding DEPs. The findings can help in our future study of the antibiotic resistance mechanism in ESBL-EC and the corresponding research and development of biomedicine-based target therapy (Fig. 10).

## Author contributions

Data curation: Bingjie Lai, Chang Tian; investigation: Jiaying Liu; project adminstration: He Ma, Yufen Jin; resources: Ke Wang; software: Bingjie Lai; supervision: Ke Wang; writing-original draft: He Ma; writing-review: Ke Wang.

## Conflicts of interest

The authors declare no conflict of interest.

## Supplementary Material

RA-010-D0RA04250A-s001

RA-010-D0RA04250A-s002

RA-010-D0RA04250A-s003

RA-010-D0RA04250A-s004

RA-010-D0RA04250A-s005

RA-010-D0RA04250A-s006

RA-010-D0RA04250A-s007

RA-010-D0RA04250A-s008

RA-010-D0RA04250A-s009

RA-010-D0RA04250A-s010
