# Supplementary figures and images for: Proteomics and metabolomics analysis reveal potential mechanism of extended-spectrum β-lactamase production in Escherichia coli
Source: RSC Adv. 2020 Jul 17;10(45):26862–73. doi: 10.1039/d0ra04250a (PMC9055503; doi:10.1039/d0ra04250a)

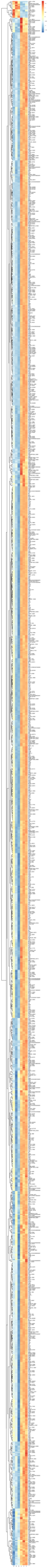

Supplement: RA-010-D0RA04250A-s008 [file RA-010-D0RA04250A-s008.pdf]
