# Supplementary material for: Proteomics and metabolomics analysis reveal potential mechanism of extended-spectrum β-lactamase production in Escherichia coli
Source: RSC Adv. 2020 Jul 17;10(45):26862–73. doi: 10.1039/d0ra04250a (PMC9055503; doi:10.1039/d0ra04250a)
Supplement: RA-010-D0RA04250A-s009 [file RA-010-D0RA04250A-s009.pdf]

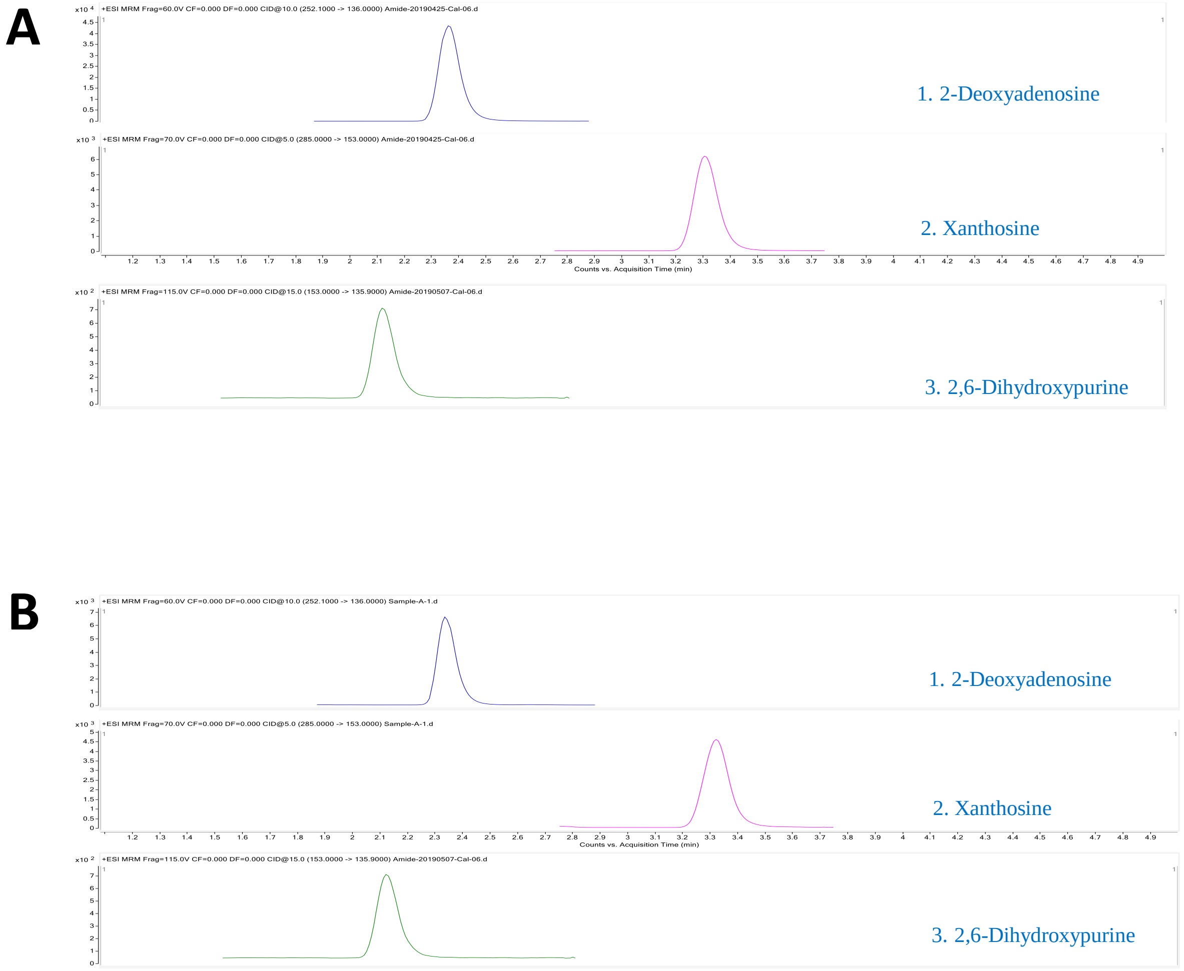

Figure S2. Representative extracted ion chromatographs (EICs) from a standard solution (A) and a sample (B) of the targeted analytes under the optimal conditions.
