# Supplementary material for: Proteomics and metabolomics analysis reveal potential mechanism of extended-spectrum β-lactamase production in Escherichia coli
Source: RSC Adv. 2020 Jul 17;10(45):26862–73. doi: 10.1039/d0ra04250a (PMC9055503; doi:10.1039/d0ra04250a)
Supplement: RA-010-D0RA04250A-s010 [file RA-010-D0RA04250A-s010.pdf]

Electronic Supplementary Material (ESI) for RSC Advances.
This journal is © The Royal Society of Chemistry 2020

High pH Reverse Phase Separation

The peptide mixture was redissolved in the buffer A (buffer A: 10 mM ammonium formate in water, pH 10.0, adjusted with ammonium hydroxide), and then fractionated by high pH separation using a Aquity UPLC system (Waters Corporation, Milford, MA) connected to a reverse phase column (BEH C_{18} column, 2.1 mm × 150 mm, 1.7 μm, 300 Å, Waters Corporation, Milford, MA). High pH separation was performed using a linear gradient. Starting from 0% B to 45% B in 35 min (B: 10 mM ammonium formate in 90% ACN, pH 10.0, adjusted with ammonium hydroxide). The column flow rate was maintained at 250 μL/min and column temperature was maintained at 45℃. Twelve fractions were collected, each fraction was dried in a vacuum concentrator for the next step.

Database Searching

Tandem mass spectra were extracted by Proteome Discoverer software (Thermo Fisher Scientific, version 1.4.0.288). Charge state deconvolution and deisotoping were not performed. All MS/MS samples were analyzed using Mascot (Matrix Science, London, UK; version 2.3). Mascot was set up to search the Uniprot database (Taxonomy: Escherichia Coli, entries 1298272) assuming the digestion enzyme trypsin. Mascot was searched with a fragment ion mass tolerance of 0.050 Da and a parent ion tolerance of 10.0 PPM. Carbamidomethyl of cysteine, TMT 6 plex of lysine and n-terminus were specified in Mascot as fixed modifications. Oxidation of methionine was specified in Mascot as a variable modification.

Quantitative Data Analysis

Use the percolator algorithm to control peptide level false discovery rates (FDR) lower than 1%. Only unique peptides were used for protein quantification, protein contains at least two unique peptides, and the method of normalization on protein median was used to correct experimental bias, the minimum number of proteins that must be observed to allow was set to 1000.

Data Preprocessing and Metabolite Annotation

MS raw data (.wiff) files were converted to the mzXML format using ProteoWizard, and processed by R package XCMS (version 3.2). The preprocessing results generated a data matrix that consisted of the retention time (RT), mass to charge ratio (m/z) values, and peak intensity. R package CAMERA was used for peak annotation after XCMS data processing, including missing value recoding and normalization with internal standard (IS). In-house MS2 database was applied in metabolites identification. 389 and 254 metabolites were detected in positive and negative ion mode, respectively. Data see in Table S6. Then the three-dimensional data were applied to SIMCA V14.1 (Sartorius Stedim Data Analytics AB, Umea, Sweden) for principal component analysis (PCA) and orthogonal projections to latent structures-discriminate analysis (OPLS-DA).

In addition, commercial databases including KEGG (http://www.genome.jp/kegg/) and MetaboAnalyst (http://www.metaboanalyst.ca/) were utilized for the pathway analysis of metabolites.

Standard Solution Preparation

Stock solutions were individually prepared by dissolving or diluting each standard substance to give a final concentration of 10 mmol/L. An aliquot of each of the stock solutions was transferred to a 10 mL

flask to form a mixed working standard solution. A series of calibration standard solutions were then prepared by stepwise dilution of this mixed standard solution.

Calibration Curves

Calibration solutions were subjected to UPLC-MRM-MS/MS analysis using the methods described above. Table S4 summarizes the results for the calibration curves where y is the peak areas for analyte, and x is the concentration (nmol/L) for analyte. Least squares method was used for the regression fitting. 1/x weighting was applied in the curve fitting since it provided highest accuracy and correlation coefficient (R^2). The level was excluded from the calibration if the signal-to-noise ratio (S/N) was close to or below 20, or accuracy of calibration was not within 80–120%. Detailed calibration curves for individual analytes are shown in Table S4.

Limit of Detection (LOD) and Limit of Quantitation (LOQ)

The calibration standard solution was diluted stepwise, with a dilution factor of 2. These standard solutions were subjected to UHPLC-MRM-MS analysis. The signal-to-noise ratios (S/N) were used to determine lower limits of quantitation (LLOQs). The LLOQs were defined as the analyte concentrations that led to peaks with signal-to-noise ratios (S/N) of 10, respectively, according to the US FDA guideline for bioanalytical method validation.

Precision and Accuracy

The precision of the quantitation was measured as the relative standard deviation (RSD), determined by injecting analytical replicates of a QC sample. The accuracy of quantitati on was measured as the analytical recovery of the QC sample determined. The percent recovery was calculated as [(mean observed concentration) / (spiked concentration)] × 100%.
